# Fat Emboli Entrapped in the Tricuspid Valve Chordae Following Autologous Fat Grafting: An Echocardiographic-Pathological Correlation

**DOI:** 10.7759/cureus.117082

**Published:** 2026-09-28

**Authors:** Thomas Langelet, Christophe Beauloye, Diego Orbegozo Cortes, Olivier Gach, Anja Kerschen

**Affiliations:** 1 Department of Cardiology, Cliniques Universitaires Saint-Luc, Brussels, BEL; 2 Department of Cardiovascular Intensive Care, Cliniques Universitaires Saint-Luc, Brussels, BEL; 3 Department of Cardiology, Clinique CHC (Centre Hospitalier Chrétien) MontLégia, Liege, BEL; 4 Department of Pathology, Cliniques Universitaires Saint-Luc, Brussels, BEL; 5 Department of Forensic Medicine, Cliniques Universitaires Saint-Luc, Brussels, BEL; 6 Pole of Morphology (MORF) Institute of Experimental and Clinical Research (IREC), UCLouvain (Université Catholique de Louvain), Brussels, BEL

**Keywords:** autologous fat grafting, disseminated intravascular coagulation, ecmo, fat embolism, liposuction, transesophageal echocardiography, tricuspid valve

## Abstract

Fat embolism is a rare but feared complication of liposuction combined with autologous fat grafting (lipofilling). It carries a high mortality rate, predominantly affecting young patients with few or no comorbidities. To date, few guidelines exist, and management remains largely supportive.

We report the case of a 40-year-old woman admitted to our center following cardiac arrest during breast lipofilling surgery. Given persistent hemodynamic instability and echocardiographic evidence of acute right ventricular overload with an intracardiac mass, veno-arterial extracorporeal membrane oxygenation (VA-ECMO) was initiated immediately upon return of spontaneous circulation. The patient subsequently developed massive disseminated intravascular coagulation (DIC), requiring 52 units of blood products within 24 hours. The clinical course was further complicated by acute respiratory distress syndrome (ARDS), recurrent DIC with stroke, and cardiogenic shock due to acute right heart failure, ultimately leading to withdrawal of care. Autopsy revealed pulmonary fat emboli and adipose material entrapped within the tricuspid valve chordae, corresponding to the intracardiac mass identified on echocardiography.

This case highlights the echocardiographic identification of a fat embolus entrapped within the tricuspid subvalvular apparatus following cosmetic fat grafting surgery. Its early recognition may reflect a substantial embolic burden and could provide additional prognostic information when interpreted alongside established clinical, hemodynamic, and echocardiographic parameters. The case also illustrates severe DIC as a potentially important systemic complication of fat embolism in this setting.

## Introduction

Fat grafting (lipofilling) is a cosmetic surgery technique involving autologous fat harvesting by lipoaspiration and reinjection into a recipient site. While most complications are minor, fat embolism remains among the most feared, resulting from the entry of adipose fragments into the venous circulation through surgically damaged vessels. This phenomenon, also well-described following orthopedic procedures and bone fractures, manifests as either macroscopic fat embolism (MAFE) or microscopic fat embolism (MIFE). MAFE is diagnosed based on radiological investigations demonstrating fatty filling defects within the vasculature, as well as on biopsy and autopsy findings. MIFE, by contrast, corresponds to fat embolism syndrome (FES), a clinical diagnosis based on an often incomplete triad of hypoxemia, cerebral involvement, and petechiae, supported by nonspecific laboratory and radiological findings [1]. Although these entities probably form part of a continuum, this distinction carries important therapeutic implications. We report a case of MAFE following breast fat grafting, complicated by pulmonary, renal, and splenic embolism. Notably, echocardiography revealed a fat embolus lodged in the tricuspid valve chordae, later confirmed at autopsy.

## Case presentation

A 40-year-old woman was admitted to the emergency department following an out-of-hospital cardiac arrest during liposuction and fat grafting surgery performed under general anesthesia at a private clinic. Her medical history was notable only for prior gastric bypass and overweight, with no chronic medications or known allergies. The cardiac arrest occurred during the fat reinjection phase, from the abdomen to the breasts. Cardiopulmonary resuscitation was initiated immediately. The first documented rhythm was asystole, followed by pulseless electrical activity. Return of spontaneous circulation was achieved after 54 minutes of cardiopulmonary resuscitation, with no preceding no-flow period and a cumulative epinephrine dose of 11 mg.

During resuscitation, bedside echocardiography revealed right ventricular dilatation with paradoxical septal motion and a mass attached to the tricuspid valve within the right ventricle (Figure 1).

**Figure 1:**
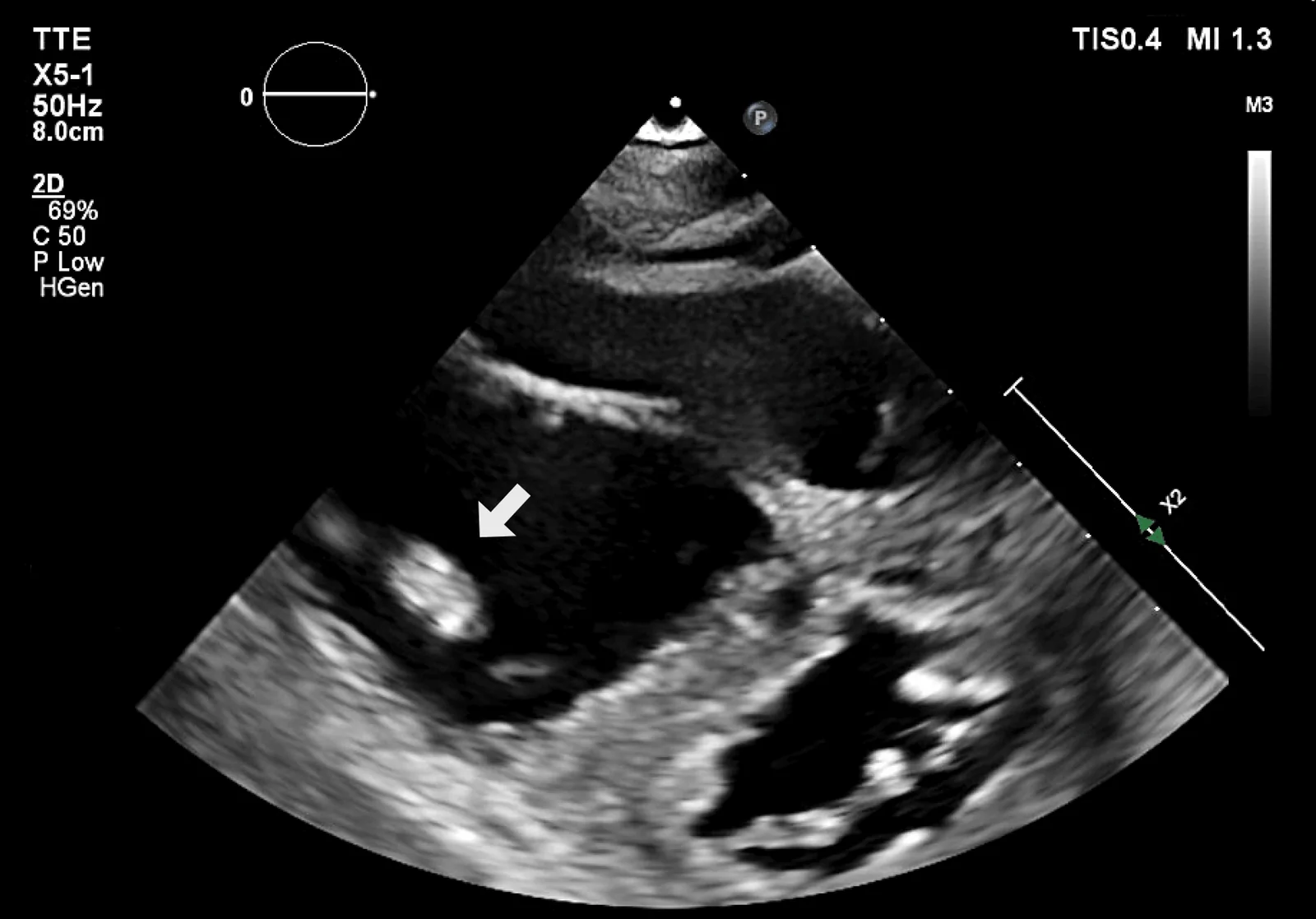
Bedside transthoracic echocardiography during cardiopulmonary resuscitation Transthoracic echocardiography demonstrates right ventricular dilatation and an intracardiac mass within the right ventricle (white arrow).

No femoral or popliteal venous obstruction was identified. Arterial blood gas analysis showed metabolic and lactic acidosis (Table 1). The electrocardiogram demonstrated sinus tachycardia with an S1Q3 pattern and incomplete right bundle branch block. Given the clinical context and echocardiographic findings, fat embolism was promptly suspected. Femoral cannulae were inserted during resuscitation in anticipation of extracorporeal membrane oxygenation (ECMO). Persistent hemodynamic instability following return of spontaneous circulation led to immediate initiation of ECMO support.

**Table 1:** Initial arterial blood gas, hematologic, and coagulation findings PaCO₂: partial pressure of arterial carbon dioxide; aPTT: activated partial thromboplastin time; PT: prothrombin time; INR: international normalized ratio; TT: thrombin time

Parameter	Result	Reference Range
Arterial blood gas analysis		
pH	7.08	7.35–7.45
PaCO₂	34 mmHg	35–45 mmHg
Bicarbonate	10 mmol/L	22–28 mmol/L
Lactate	10.3 mmol/L	0.5–2.2 mmol/L
Hematology and coagulation		
Hemoglobin	5.6 g/dL	12.2–15.0 g/dL
Platelet count	46 ×10⁹/L	150–450 ×10⁹/L
aPTT	>180 s	25.1–38.5 s
PT	≥100 s	9.35–14.30 s
INR	>7.00	0.80–1.20
TT	>120 s	10.0–18.0 s
Fibrinogen	72 mg/dL	150–450 mg/dL
D-dimer	10,299 ng/mL	≤500 ng/mL

Transesophageal echocardiography demonstrated a mobile mass entrapped within the tricuspid valve chordae, with mobility documented on Video 1.

**Video 1:** Transesophageal echocardiography showing a mobile mass entrapped within the tricuspid subvalvular apparatus Transesophageal echocardiography demonstrating a mobile mass entrapped within the tricuspid subvalvular apparatus.

Computed tomography (CT) angiography revealed segmental and subsegmental pulmonary embolism in the right lower lobe (Figure 2A), as well as splenic and renal emboli. Concurrently, massive hemorrhage was observed through the trocar insertion sites. Laboratory findings demonstrated severe anemia and disseminated intravascular coagulation (DIC) (Table 1), yielding an International Society on Thrombosis and Haemostasis DIC score of 8/8 [2]. A massive transfusion protocol was activated, with administration of 31 units of packed red blood cells, 12 units of fresh frozen plasma, nine platelet pools, and 6 g of fibrinogen overnight.

**Figure 2:**
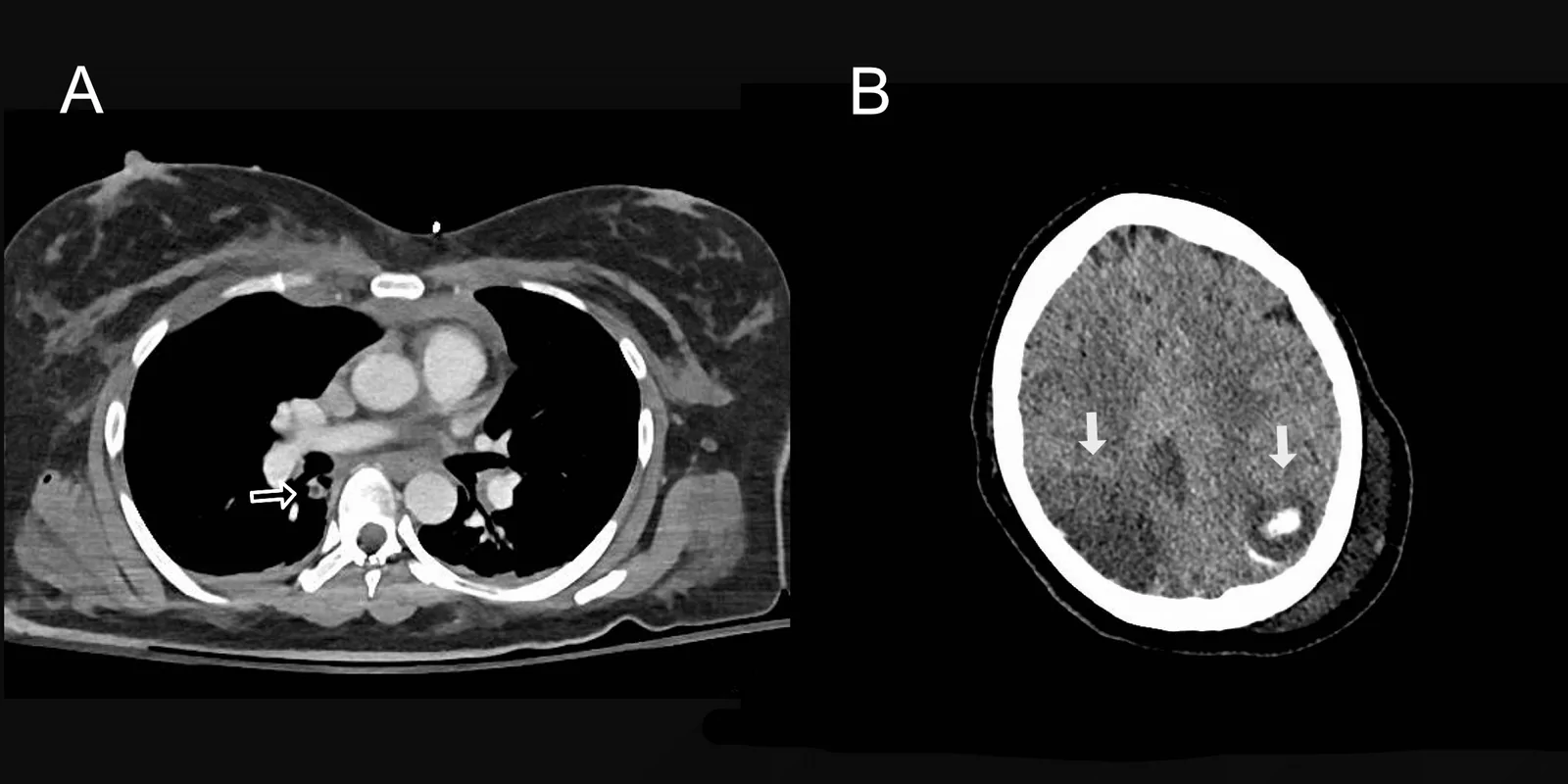
Computed tomography findings during hospitalization Panel A: CT pulmonary angiography performed on admission demonstrating a pulmonary arterial filling defect in the right lower lobe (white arrow). Panel B: Brain CT performed on day 4 demonstrating bilateral posterior ischemic lesions with hemorrhagic transformation (white arrows).

Over the following days, coagulation partially improved, and heparin was initiated on day 2. However, DIC remained active, evidenced by persistently deranged coagulation parameters, recurrent ECMO oxygenator thrombosis, epistaxis requiring packing, and iterative transfusions. On day 4, brain CT demonstrated bilateral posterior ischemic lesions with hemorrhagic transformation (Figure 2B). Management included optimization of systemic parameters to limit secondary brain injury (blood pressure, oxygenation, carbon dioxide, and glycemic control), along with closer monitoring of ECMO anticoagulation, targeting an activated partial thromboplastin time (aPTT) at the lower end of the therapeutic range to balance hemorrhagic risk with the need to maintain circuit patency.

Severe hypoxemia developed due to acute respiratory distress syndrome (ARDS), progressing despite maximal ECMO support, optimized mechanical ventilation, and targeted antibiotic therapy for pulmonary superinfection. On day 7, a jugular venous cannula was placed for conversion to veno-arteriovenous (V-AV) ECMO, which temporarily improved oxygenation. Conversion to veno-venous (VV) ECMO on day 12 was followed by acute hemodynamic deterioration with oxygenator failure, acute right heart failure, and severe peripheral hypoperfusion, necessitating reinstitution of V-AV ECMO. Subsequent imaging revealed an acute left middle cerebral artery ischemic stroke and worsening pulmonary consolidations. Together with a marked increase in D-dimer levels, persistent coagulation abnormalities, and recurrent thrombotic complications, these findings were consistent with ongoing uncontrolled disseminated intravascular coagulation.

In consultation with the family, withdrawal of care was decided on day 13 given irreversible multi-organ failure.

A medical autopsy revealed multiple macroscopically visible fat emboli within both peripheral and large peribronchial pulmonary vessels (Figure 3A). Histopathology demonstrated infarcted parenchyma, diffuse alveolar damage, and focal purulent superinfection with dense neutrophilic infiltrate (Figure 3B). The lungs were markedly congested, weighing 1,100 g (left) and 1,080 g (right).

**Figure 3:**
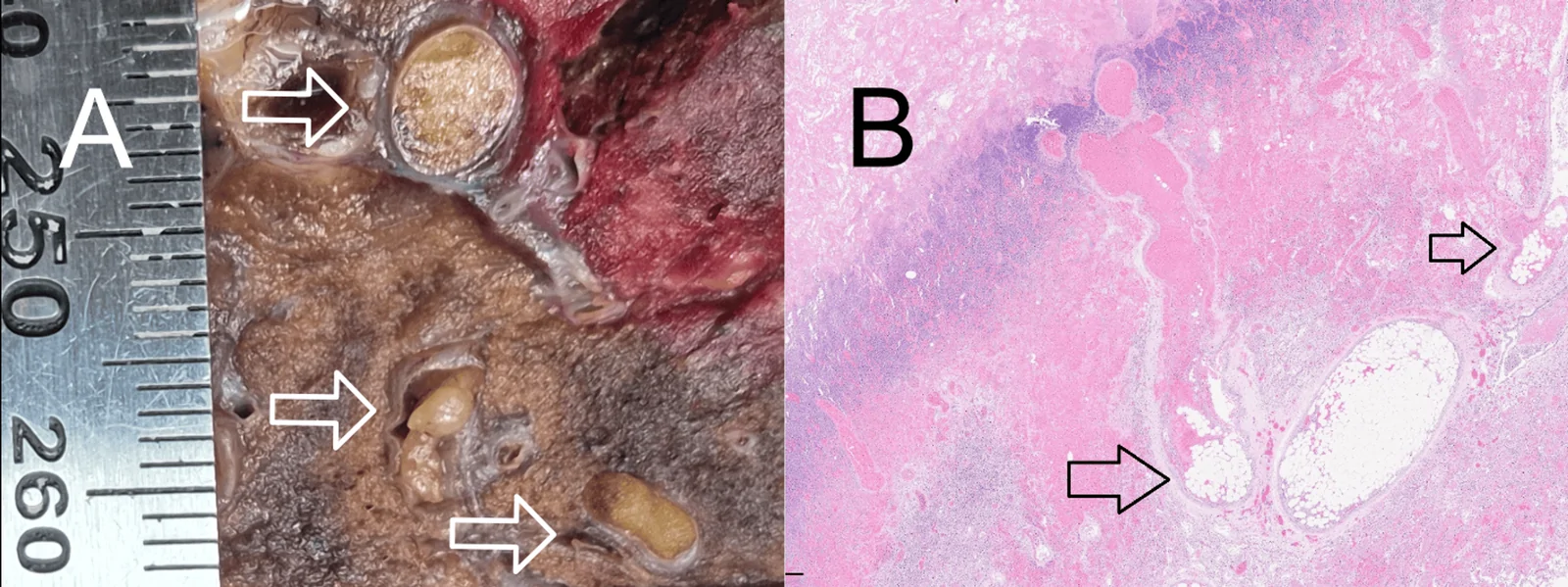
Gross and histopathological findings of pulmonary fat embolism Panel A: Multiple fat emboli within the pulmonary vasculature on gross examination (white arrows). Panel B: Histopathological examination showing intravascular adipose tissue fragments (black arrows), with associated pulmonary infarction and focal suppuration. Hematoxylin and eosin stain, 1.25× magnification.

The heart was of normal size (340 g) with anatomically normal chambers. The intracardiac mass previously identified by transesophageal echocardiography (Figure 4A) corresponded to adipose material found entrapped within the tricuspid valve chordae at autopsy (Figure 4B). Histopathological examination subsequently confirmed the adipose nature of the material (Figure 4C).

**Figure 4:**
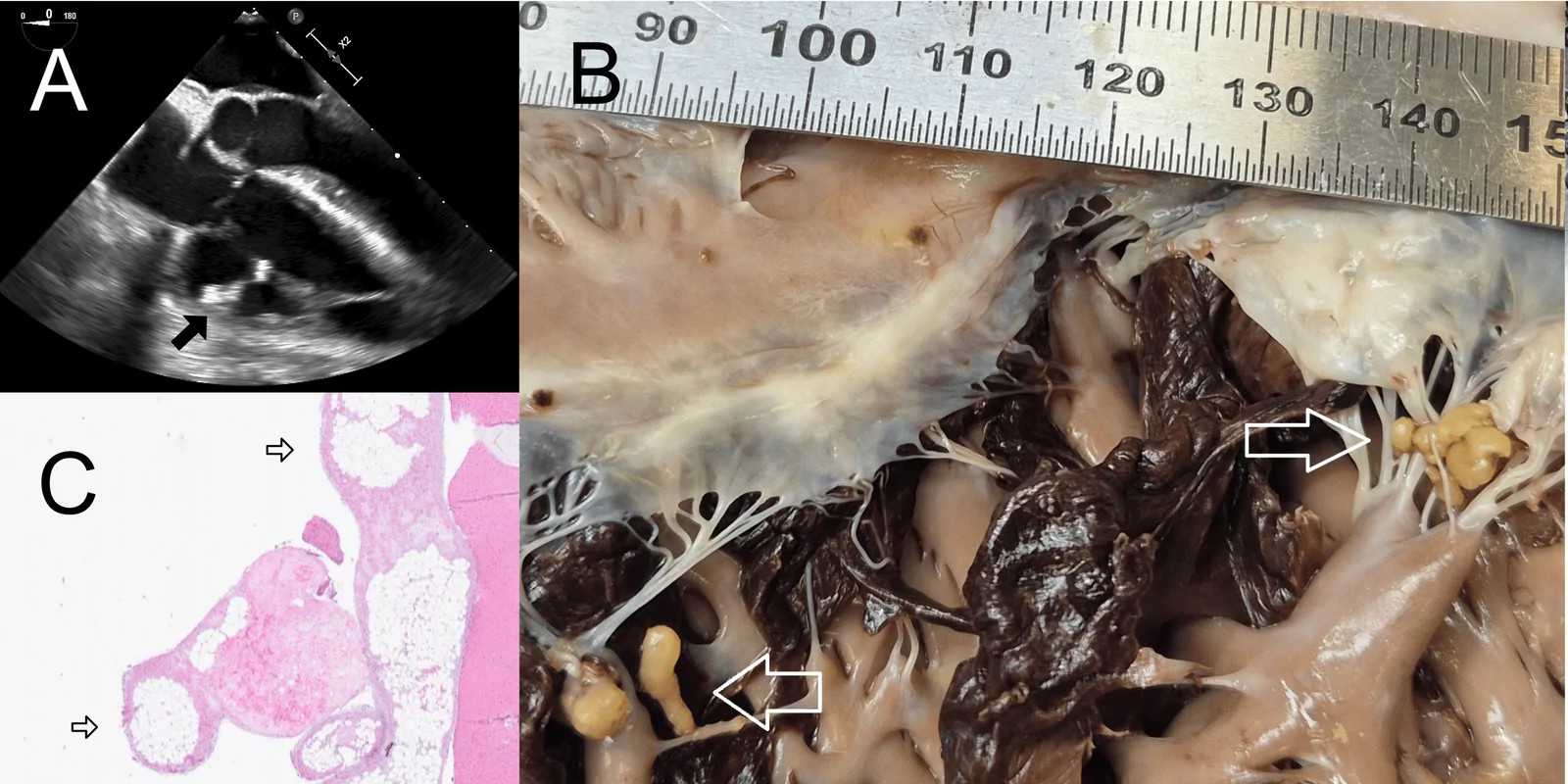
Echocardiographic–pathological correlation of fat emboli entrapped within the tricuspid valve chordae Panel A: Transesophageal echocardiography demonstrating a mobile mass entrapped within the tricuspid valve chordae (black arrow). Panel B: Macroscopically visible fat fragments within the chordae tendineae of the tricuspid valve (white arrows). Panel C: Adipose tissue fragments entrapped within the tricuspid valve chordae confirmed histopathologically (black arrows). Hematoxylin and eosin stain, 0.86× magnification.

## Discussion

A systematic review reported a mortality rate of 86.1% among 36 published cases of pulmonary fat embolism following fat grafting [3]. A notable feature of fat embolism in cosmetic surgery is the rapid onset of symptoms compared to the 24-72-hour delay typically observed after fractures or orthopedic procedures [3,4]. Furthermore, early symptom onset appears to correlate with greater clinical severity, including higher rates of mechanical ventilation, cardiac arrest, and mortality [4].

ECMO may provide temporary cardiopulmonary support in selected patients with refractory hemodynamic or respiratory failure. Wolfe et al. reported the case of a 28-year-old patient with MAFE following gluteal fat grafting who was placed on ECMO owing to resistance to vasopressor and ventilatory support. The patient was successfully weaned off after six days and subsequently discharged home after 24 days [5]. In our case, ECMO was initiated immediately after return of spontaneous circulation because of persistent hemodynamic instability. The echocardiographic finding of an intracardiac fat embolus with acute right heart failure was, in retrospect, consistent with the overall severity of the presentation, which was subsequently compounded by hemorrhagic shock.

Although intracardiac fat should not by itself be considered an indication for extracorporeal support, its visualization may reflect a substantial embolic burden and could serve as an additional marker of severity, contributing, alongside established clinical, hemodynamic, and echocardiographic parameters, to early risk stratification. Decisions regarding extracorporeal support should nevertheless remain based on conventional indications.

Two aspects of this case are particularly noteworthy. First, the intracardiac fat embolus was identified by echocardiography and subsequently confirmed at autopsy, providing a direct echocardiographic-pathological correlation. In addition to providing an early diagnostic clue, this finding may represent a potential marker of disease severity. Reports of intracardiac fat following cosmetic fat grafting remain scarce, with, for example, right ventricular fat identified at autopsy after gluteal fat grafting in a 25-year-old in Colombia [6] and a 29-year-old in the United States [7], both of whom died perioperatively. Second, to our knowledge, DIC has rarely been reported as a complication of liposuction or fat grafting. Hematologic abnormalities (anemia, thrombocytopenia, and elevated D-dimers) have been described in this setting [3,4], though without reaching critical thresholds. By contrast, DIC is a well-recognized complication of fat embolism in orthopedic settings [8,9] mediated by free fatty acid-induced coagulation activation and endothelial injury. The underlying pathophysiological mechanism is likely similar; its apparent rarity after cosmetic surgery may reflect the low and underreported prevalence of fat embolism in this context, together with early clinical deterioration and high initial mortality, which may preclude timely documentation of DIC.

A limitation of this report is the absence of both an ocular assessment and a dedicated cerebral examination at autopsy to document fat emboli in these territories. Furthermore, cerebral magnetic resonance imaging (MRI), more sensitive than CT for detecting cerebral fat embolism, was precluded by ECMO.

From a public health perspective, recommendations are needed to prevent potentially life-threatening complications of these aesthetic procedures in young, often otherwise healthy patients. Such efforts have already been undertaken by the Aesthetic Surgery Education and Research Foundation (ASERF) for gluteal fat grafting, drawing on an anonymous survey of 692 plastic surgeons to develop recommendations aimed at improving procedural safety [10]. However, the rarity of fat embolism, together with operative reports that are often limited or unavailable, hampers the development of specific technical recommendations. Equally important, these procedures should be performed within or in close proximity to facilities capable of managing potentially life-threatening complications.

## Conclusions

Fat embolism is a severe and potentially fatal complication of liposuction and fat grafting. Echocardiographic visualization of an intracardiac fat embolus may reflect a substantial embolic burden and, when interpreted alongside established clinical, hemodynamic, and echocardiographic parameters, could provide additional prognostic information. Such a finding should not, however, be considered an indication for extracorporeal support in isolation, and therapeutic decisions should remain guided by established management algorithms. Finally, the severe disseminated intravascular coagulation observed in this case underscores the importance of close laboratory monitoring for this potentially serious complication.
